# Supplementary material for: Fe/Mg-Modified Carbonate Apatite with Uniform Particle Size and Unique Transport Protein-Related Protein Corona Efficiently Delivers Doxorubicin into Breast Cancer Cells
Source: Nanomaterials (Basel). 2020 Apr 27;10(5):834. doi: 10.3390/nano10050834 (PMC7712760; doi:10.3390/nano10050834)
Supplement: Supplementary file 1 [file nanomaterials-10-00834-s001.pdf]

nanomaterials

MDPI

Fe/Mg-Modified Carbonate Apatite with Uniform Particle Size and Unique Transport Protein-Related Protein Corona Efficiently Delivers Doxorubicin into Breast Cancer Cells

Sheikh Tanzina Haque [1], Md. Emranul Karim [1], Syafiq Asnawi Zainal Abidin [1], Iekhsan Othman [1], Mark M. Banaszak Holl [2] and Ezharul Hoque Chowdhury [1,2,3,*]

Supplemental Materials:

A. **CA**

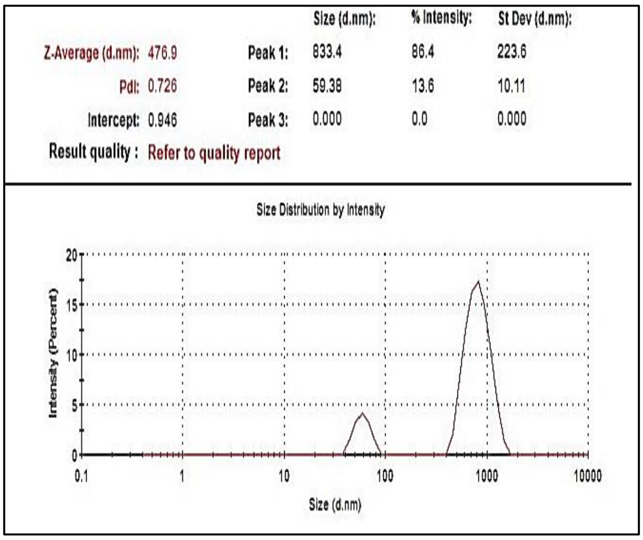

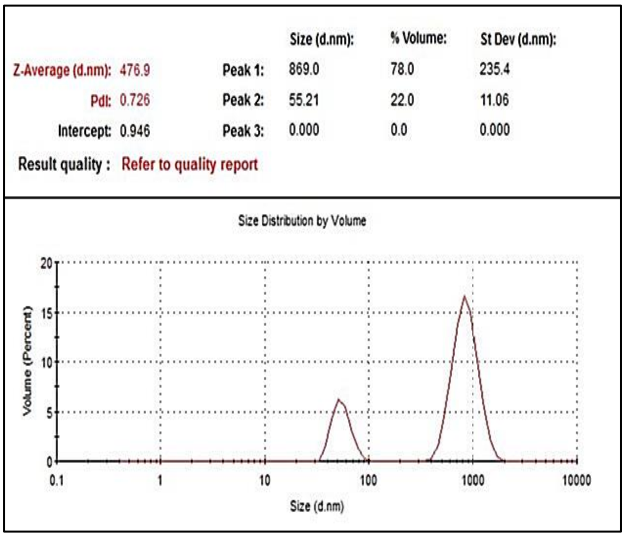

B. **Low Fe/Mg-CA**

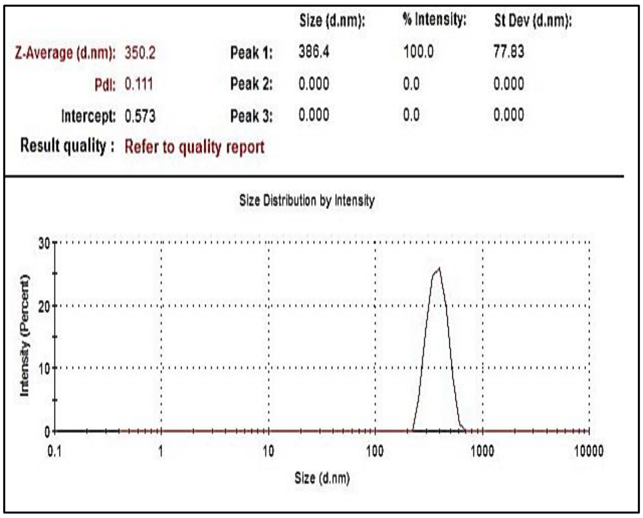

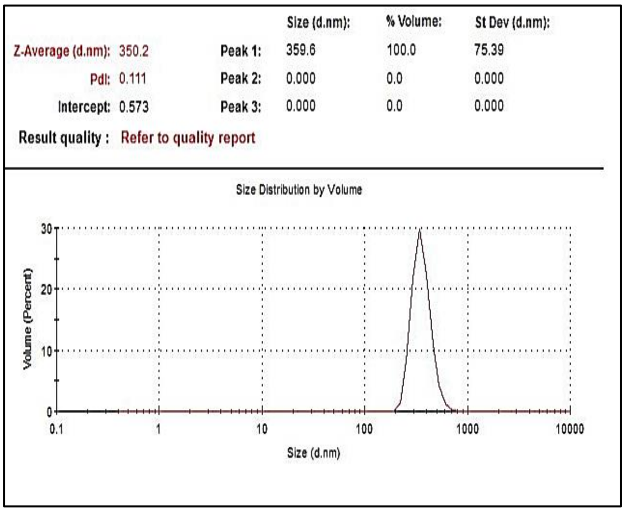

C. **High Fe/Mg-CA**

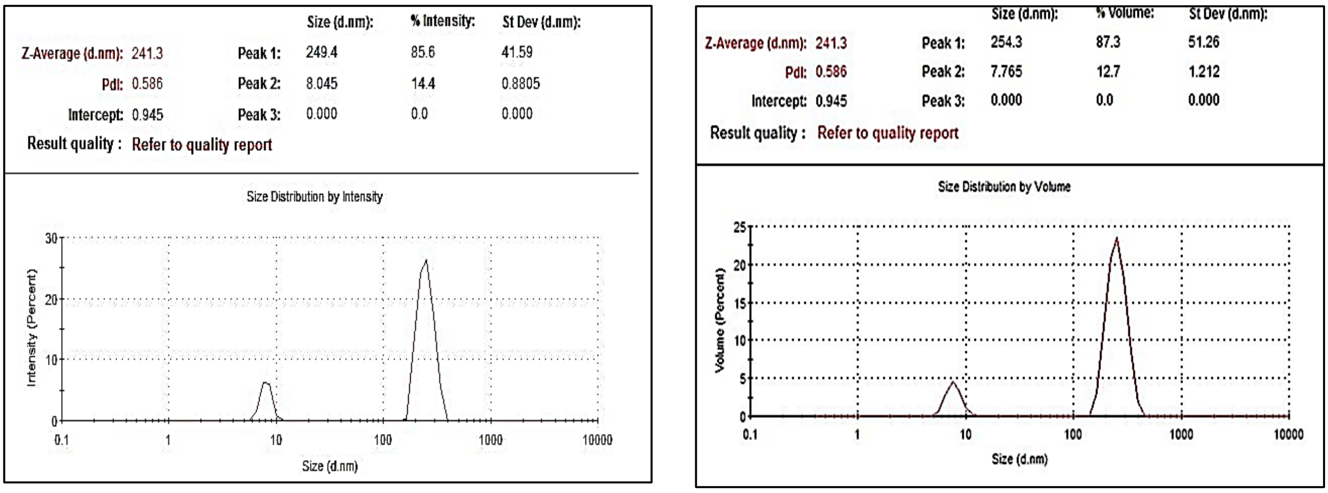

Figure S1: Representative data for dynamic light scattering technique used to demonstrate the particle size distribution by intensity and volume for CA and Fe/Mg-CA NPs. (A) Size distribution by intensity and volume for CA NPs. (B) Size distribution by intensity and volume for low Fe/Mg-CA NPs. (C) Size distribution by intensity and volume for high Fe/Mg-CA NPs.

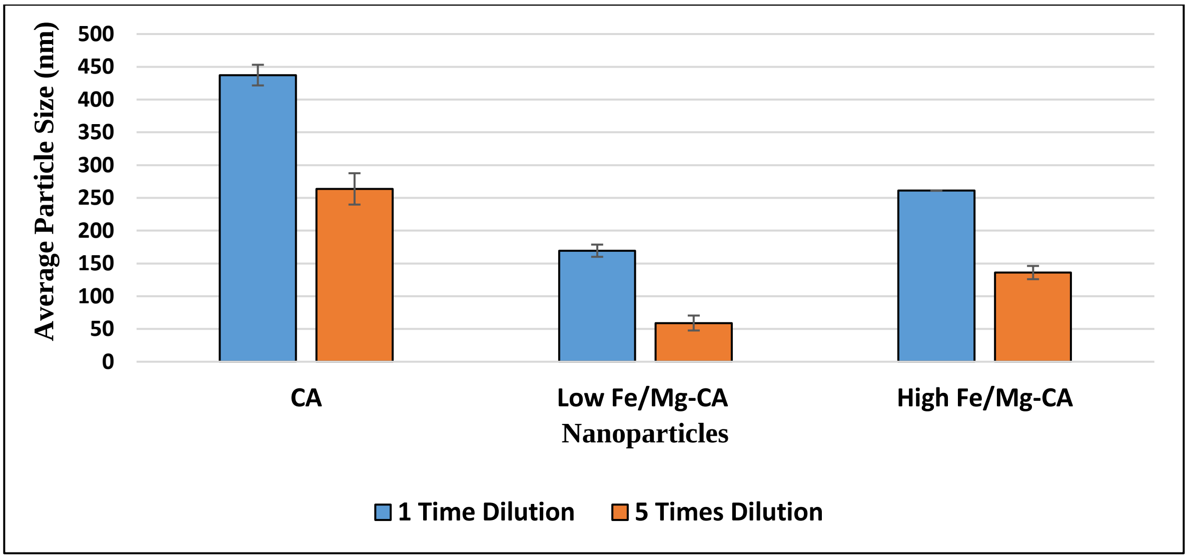

Figure S2: Comparison of z-average diameter of 1× and 5× diluted samples of CA, low Fe/Mg-CA and high Fe/Mg-CA NPs.

A. CA

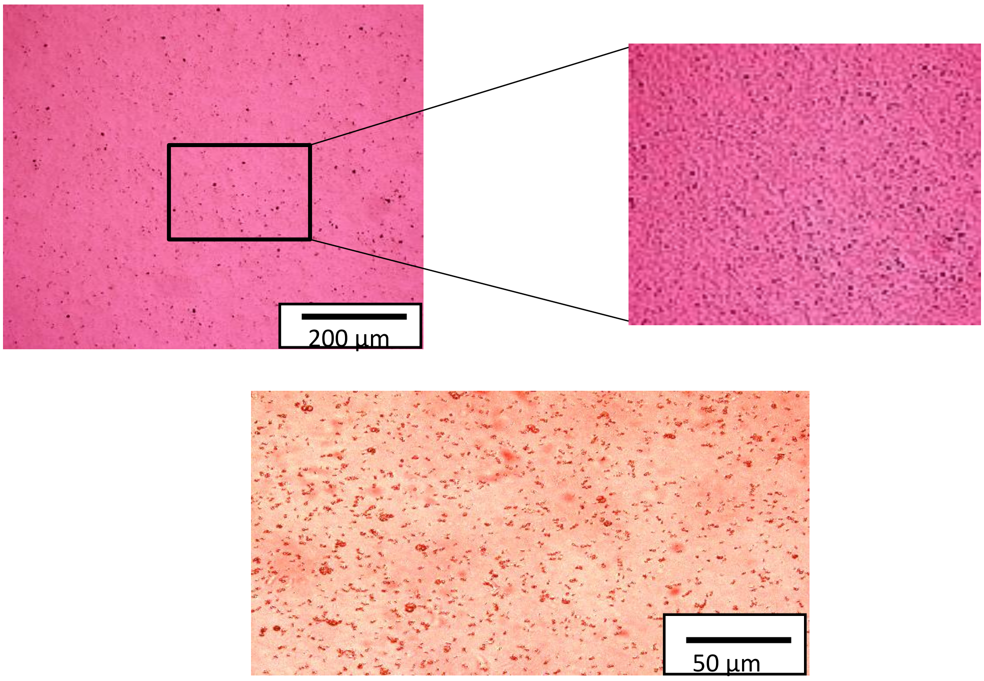

B. Low Fe/Mg-CA

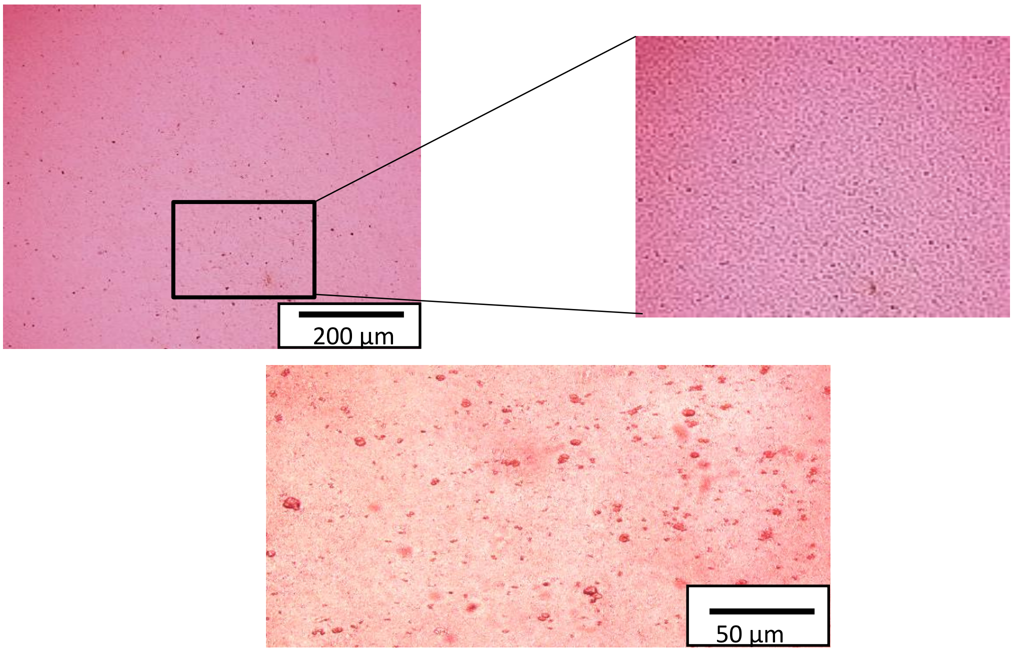

C. High Fe/Mg-CA

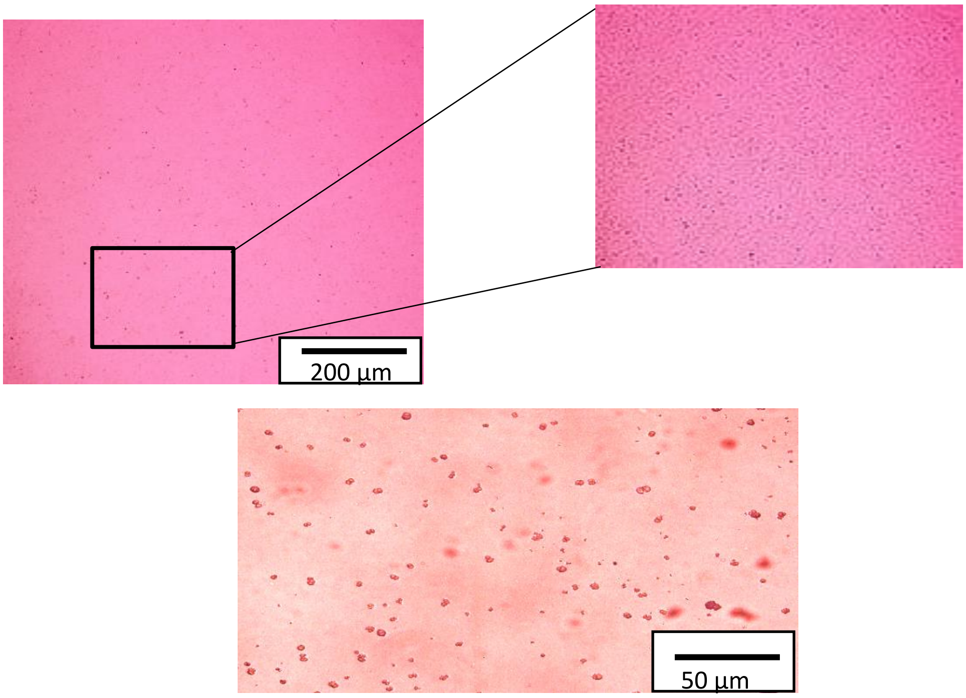

Figure S3: Observation of particle aggregates by optical microscopy with different magnifications, i.e. 40× (scale bar of 200 µm), 10× (scale bar of 50 µm) in presence of 10% FBS after 1 hour incubation. (A) CA, (B) Low Fe/Mg-CA and (C) High Fe/Mg-CA.

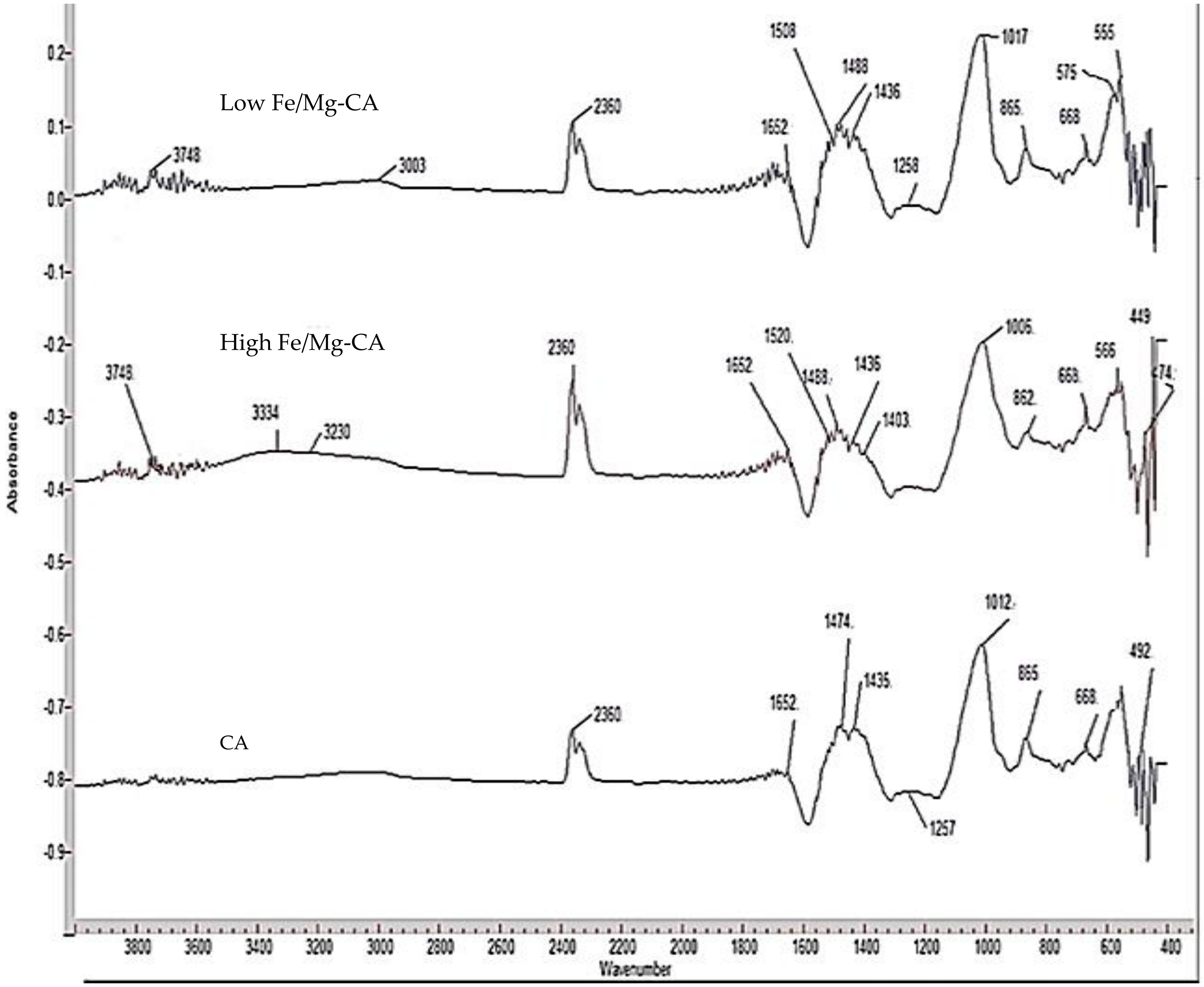

Figure S4: FTIR spectra for CA formulated with 40 mM Ca^{2+}, low Fe/Mg-CA generated with 40 mM Ca^{2+}, 10 μM Fe^{3+} and 40 mM Mg^{2+}, and high Fe/Mg-CA synthesized with 40 mM Ca^{2+}, 50 μM Fe^{3+} and 120 mM Mg^{2+}. The change in formulation was done in order to obtain significant amount of lyophilized powder for the analysis.

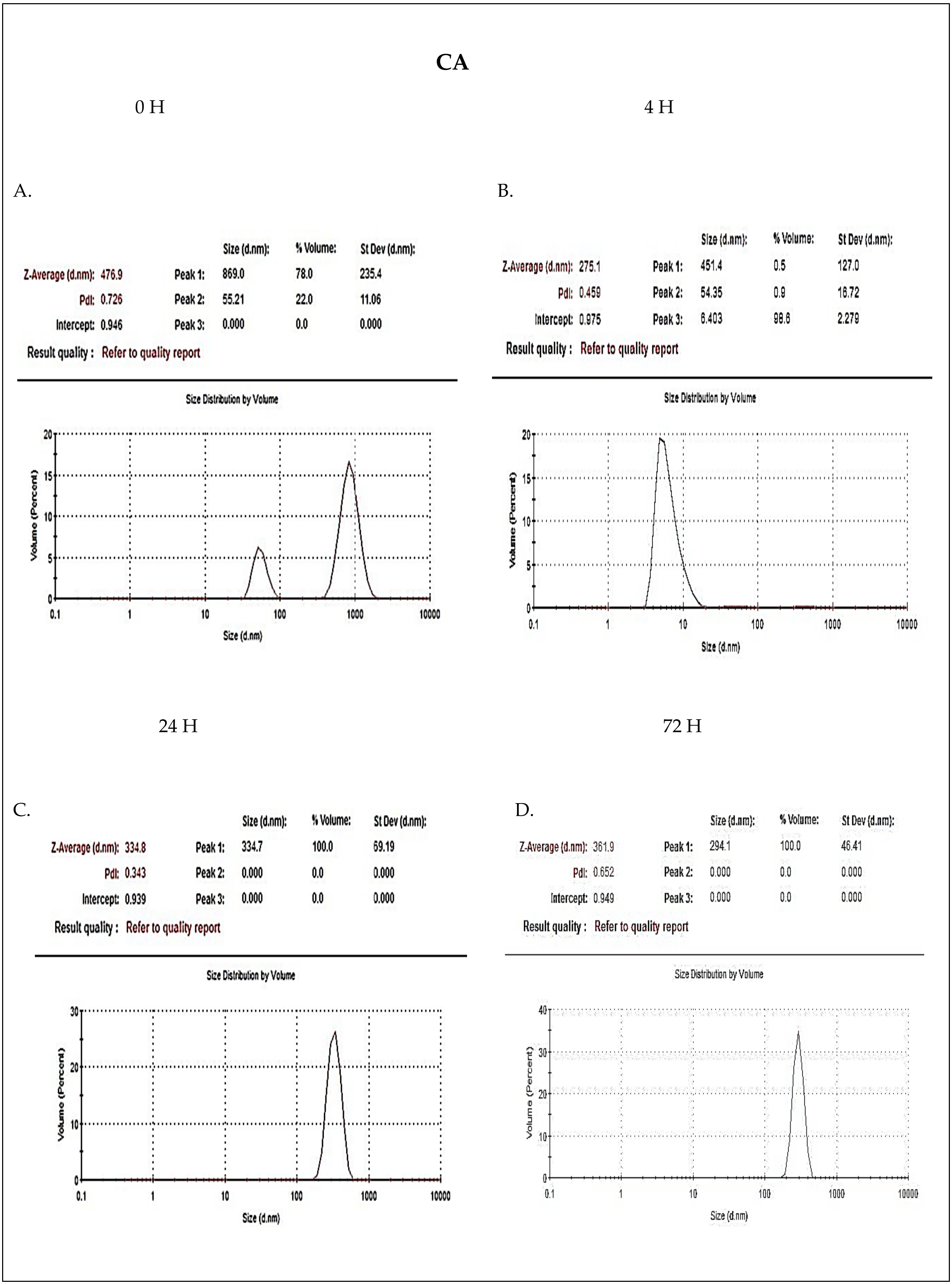

CA
0 H
4 H
A.
Size (d.nm): % Volume: St Dev (d.nm):
Z-Average (d.nm): 476.9 Peak 1: 869.0 78.0 235.4
PdI: 0.726 Peak 2: 55.21 22.0 11.06
Intercept: 0.946 Peak 3: 0.000 0.0 0.000
Result quality : Refer to quality report
Size Distribution by Volume
Volume (Percent)
Size (d.nm)
B.
Size (d.nm): % Volume: St Dev (d.nm):
Z-Average (d.nm): 275.1 Peak 1: 451.4 0.5 127.0
PdI: 0.459 Peak 2: 54.35 0.9 16.72
Intercept: 0.975 Peak 3: 6.403 98.6 2.279
Result quality : Refer to quality report
Size Distribution by Volume
Volume (Percent)
Size (d.nm)
24 H
72 H
C.
Size (d.nm): % Volume: St Dev (d.nm):
Z-Average (d.nm): 334.8 Peak 1: 334.7 100.0 69.19
PdI: 0.343 Peak 2: 0.000 0.0 0.000
Intercept: 0.939 Peak 3: 0.000 0.0 0.000
Result quality : Refer to quality report
Size Distribution by Volume
Volume (Percent)
Size (d.nm)
D.
Size (d.nm): % Volume: St Dev (d.nm):
Z-Average (d.nm): 361.9 Peak 1: 294.1 100.0 46.41
PdI: 0.652 Peak 2: 0.000 0.0 0.000
Intercept: 0.949 Peak 3: 0.000 0.0 0.000
Result quality : Refer to quality report
Size Distribution by Volume
Volume (Percent)
Size (d.nm)

Low Fe/Mg-CA

0 H

E.

			Size (d.nm):	% Volume:	St Dev (d.nm):
Z-Average (d.nm):	350.2	Peak 1:	359.6	100.0	75.39
PdI:	0.111	Peak 2:	0.000	0.0	0.000
Intercept:	0.573	Peak 3:	0.000	0.0	0.000

Result quality : Refer to quality report

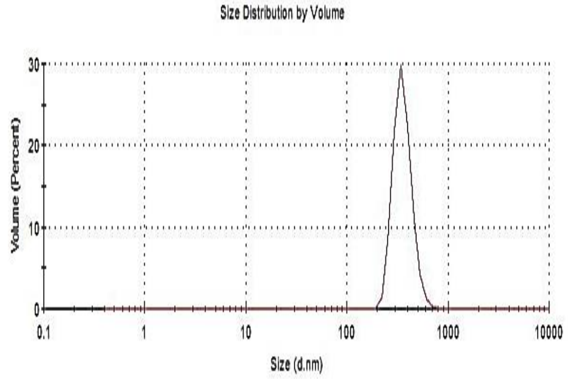

4 H

F.

			Size (d.nm):	% Volume:	St Dev (d.nm):
Z-Average (d.nm):	223.5	Peak 1:	273.0	0.1	88.54
PdI:	0.412	Peak 2:	17.98	3.9	3.904
Intercept:	0.979	Peak 3:	5.639	96.0	1.188

Result quality : Refer to quality report

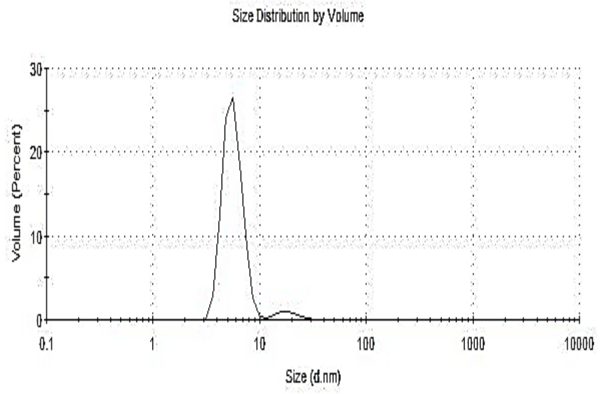

24 H

G.

			Size (d.nm):	% Volume:	St Dev (d.nm):
Z-Average (d.nm):	296.4	Peak 1:	234.9	100.0	42.79
PdI:	0.031	Peak 2:	0.000	0.0	0.000
Intercept:	0.971	Peak 3:	0.000	0.0	0.000

Result quality : Refer to quality report

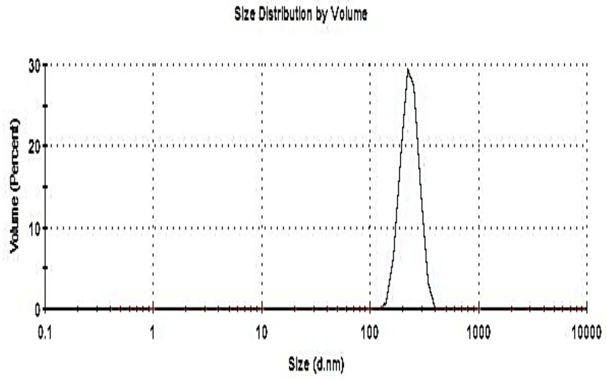

72 H

H.

			Size (d.nm):	% Volume:	St Dev (d.nm):
Z-Average (d.nm):	374.9	Peak 1:	440.0	100.0	76.14
PdI:	0.264	Peak 2:	0.000	0.0	0.000
Intercept:	0.947	Peak 3:	0.000	0.0	0.000

Result quality : Refer to quality report

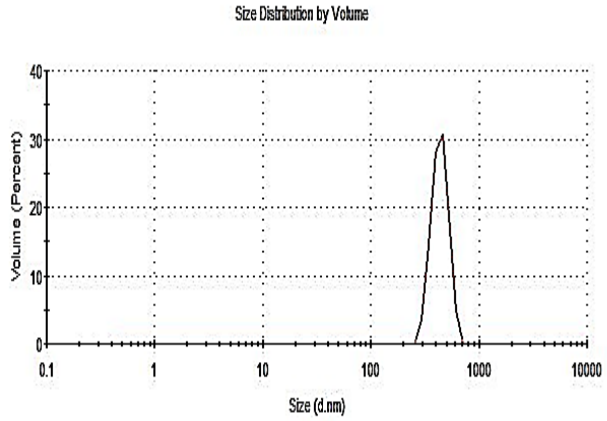

High Fe/Mg-CA

0 Hour

I.

			Size (d.nm):	% Volume:	St Dev (d.nm):
Z-Average (d.nm):	241.3	Peak 1:	254.3	87.3	51.26
PdI:	0.586	Peak 2:	7.765	12.7	1.212
Intercept:	0.945	Peak 3:	0.000	0.0	0.000

Result quality : Refer to quality report

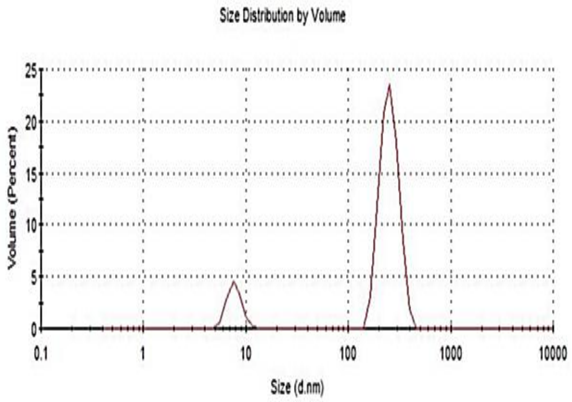

4 H

J.

			Size (d.nm):	% Volume:	St Dev (d.nm):
Z-Average (d.nm):	197.1	Peak 1:	332.7	0.1	106.9
PdI:	0.380	Peak 2:	124.2	0.0	26.58
Intercept:	0.974	Peak 3:	16.37	5.3	3.770

Result quality : Refer to quality report

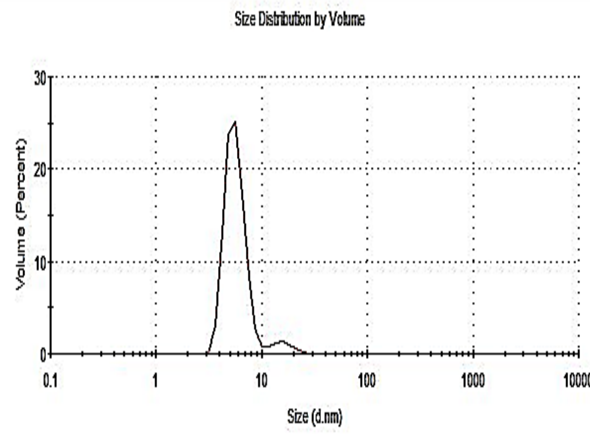

24 H

K.

			Size (d.nm):	% Volume:	St Dev (d.nm):
Z-Average (d.nm):	347.9	Peak 1:	335.4	100.0	66.78
PdI:	0.353	Peak 2:	0.000	0.0	0.000
Intercept:	0.964	Peak 3:	0.000	0.0	0.000

Result quality : Refer to quality report

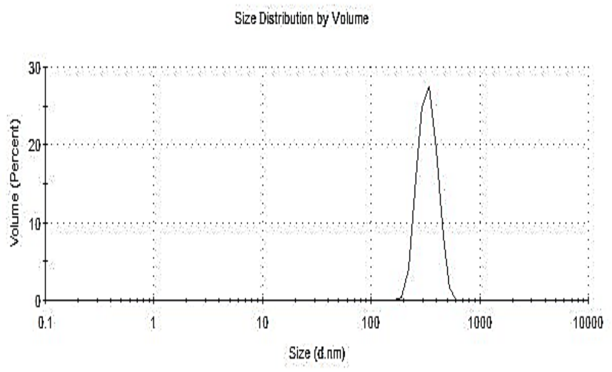

72 H

L.

			Size (d.nm):	% Volume:	St Dev (d.nm):
Z-Average (d.nm):	380.7	Peak 1:	454.8	100.0	84.55
PdI:	0.312	Peak 2:	0.000	0.0	0.000
Intercept:	0.950	Peak 3:	0.000	0.0	0.000

Result quality : Refer to quality report

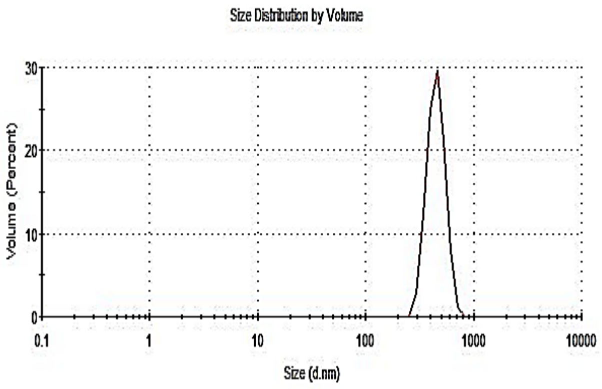

Figure S5: Representative data for dynamic light scattering technique used to demonstrate the PDI value and particle size distribution by volume for CA and Fe/Mg-CA NPs. (A) CA at 0 h. (B) CA at 4 h. (C) CA at 24 h. (D) CA at 72 h. (E) Low Fe/Mg-CA at 0 h. (F) Low Fe/Mg-CA 4 h. (G) Low Fe/Mg-CA at 24 h. (H) Low Fe/Mg-CA at 72 h. (I) High Fe/Mg-CA at 0 h. (J) High Fe/Mg-CA at 4 h. (K) High Fe/Mg-CA at 24 h. (L) High Fe/Mg-CA at 72 h.

The representative data for the elemental analysis of CA, low Fe/Mg-CA and high Fe/Mg-CA were presented below-

A. CA

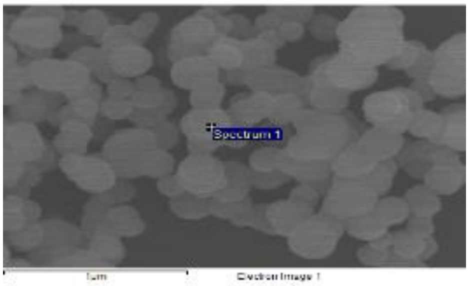

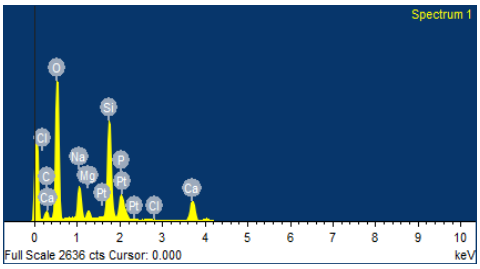

Table S1: Elemental analysis for CA.

Element	Weight %	Atomic %
Ca	11.67	12.76
Mg	1.09	0.94
C	5.04	8.86
P	4.24	2.89
O	47.38	62.55
Si	16.97	12.76
Pt	8.08	0.87
Na	5.16	4.74
Cl	0.34	0.22
Total	100	

B. Low Fe/Mg-CA

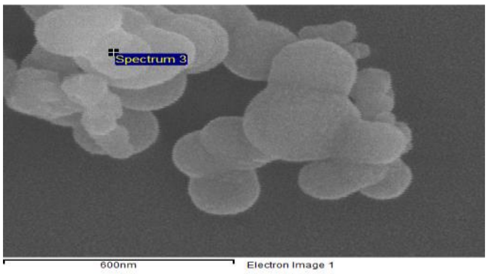

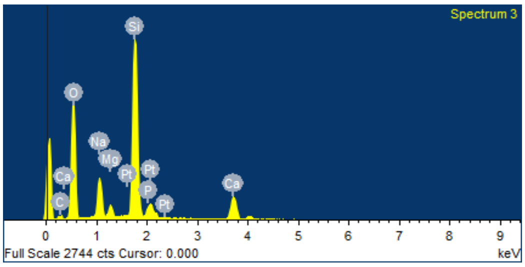

Table S2: Elemental analysis for low Fe/Mg-CA.

Element	Weight %	Atomic %
Ca	6.71	3.39
Mg	1.55	1.29
C	4.57	7.70
P	1.46	0.95
O	49.49	62.58
Si	24.48	17.63
Pt	4.98	0.52
Na	6.75	5.94
Total	100	

C. High Fe/Mg-CA

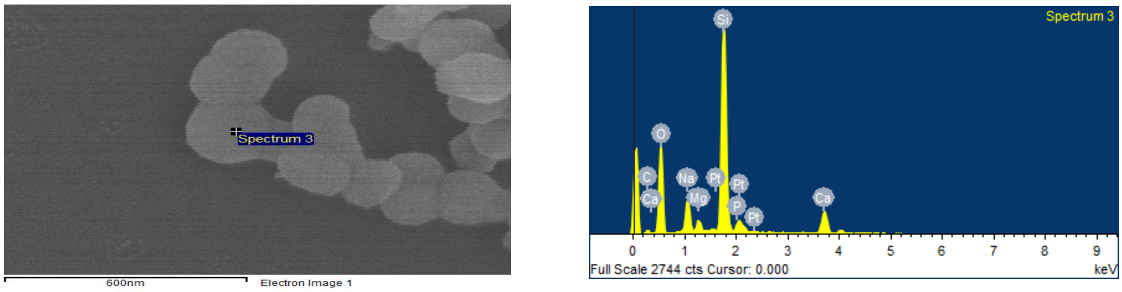

Table S3: Elemental analysis for high Fe/Mg-CA.

Element	Weight %	Atomic %
Ca	7.1	3.69
Mg	1.74	1.49
C	4.44	7.69
P	1.24	0.83
O	44.6	58.03
Si	30.47	22.58
Pt	4.69	0.5
Na	5.73	5.19
Total	100	

Figure S6: Representative data for the elemental analysis for (A) CA (B) Low Fe/Mg-CA (C) High Fe/Mg-CA.

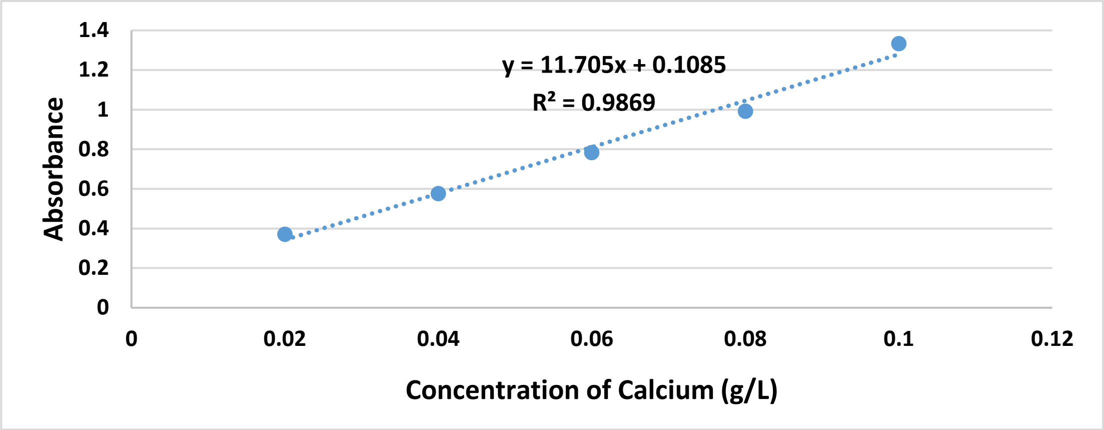

Figure S7: Standard curve for Calcium.

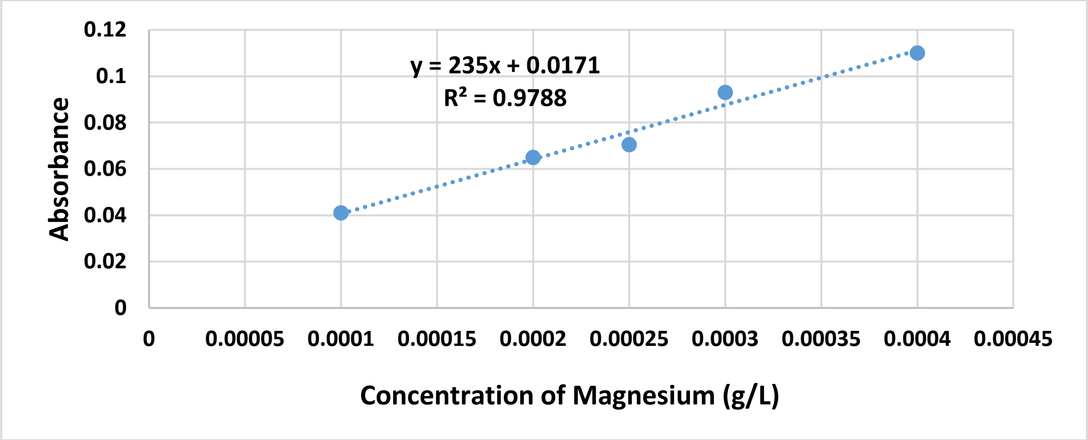

Figure S8: Standard curve for Magnesium.

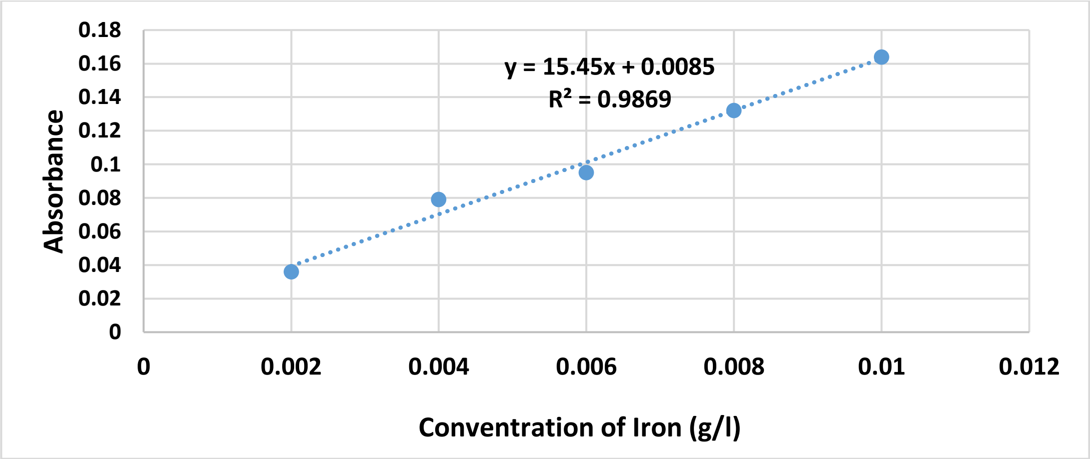

Figure S9: Standard curve for Iron.

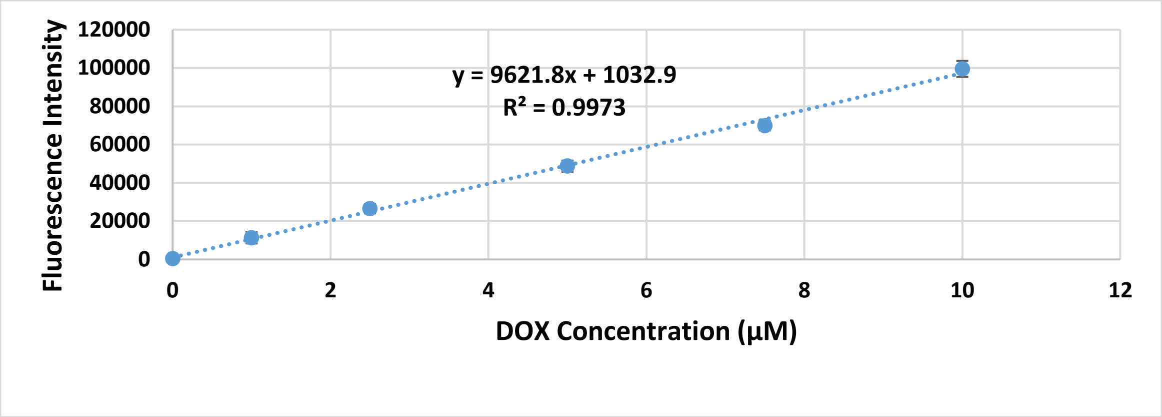

Figure S10: Standard Curve for free DOX at different concentrations.

Table S4: Cytotoxicity enhancement (%) of DOX-CA and DOX-Fe/Mg-CA NPs by thiazolyl blue tetrazolium bromide (MTT) assay in Michigan Cancer Foundation-7 (MCF-7) cells after 48 h of treatment.

NPs with different concentrations of DOX	Cytotoxicity enhancement (%) in MCF-7 cells
CA+DOX 1 nM	1± 0.45
CA+DOX 10 nM	3 ± 0.38
CA+DOX 100 nM	2 ± 0.29
CA+DOX 1μM	11± 0.81
Low Fe/Mg-CA+DOX 1 nM	3± 0.48
Low Fe/Mg-CA+DOX 10 nM	5± 0.79
Low Fe/Mg-CA+DOX 100 nM	15± 0.29
Low Fe/Mg-CA+DOX 1μM	11± 0.82
High Fe/Mg-CA+DOX 1 nM	2± 0.48
High Fe/Mg-CA+DOX 10 nM	6± 0.38
High Fe/Mg-CA+DOX 100 nM	12± 0.28
High Fe/Mg-CA+DOX 1μM	13± 0.44

Table S5. List of identified proteins with CA in 10% FBS along with their isoelectric point (pI) and major biological functions.

Functional classification of protein	Identified Protein	pI	Detailed Function
**Transport Protein**	ALB protein	5.7	1. Zn^{2+} binding
	Serum Albumin	5.53	1. Transport<br>2. Lipid binding<br>3. Metal binding<br>4. Bilirubin binding<br>5. Helps maintain osmolarity of blood<br>6. Dysopsonin
	Alpha-2-HS glycoprotein	5.94	1. Enhance endocytosis<br>2. Ca^{2+} binding<br>3. Post-translational protein modification<br>4. Protein metabolism
	cDNA FLJ51509	7.42	1. Behaves similar to Alpha-fetoprotein, i.e- it takes part in metal, fatty acids and bilirubin binding, involved in cellular protein and progesterone metabolism
	Transthyretin	5.6	1. Thyroid hormone binding<br>2. Identical protein binding<br>3. Transport iron<br>4. Transport thyroxine to the brain<br>5. Cellular protein metabolism<br>6. Neutrophil degranulation<br>7. Retinol metabolism
	Serotransferrin	6.81	1. Fe^{3+} binding<br>2. Bicarbonate binding<br>3. Transport iron
	Transferrin variant	6.68	1. Fe^{3+} binding<br>2. Transport Fe^{3+} within transmembrane<br>3. Involved in iron ion homeostasis in cells
	Transferrin	6.97	1. Fe^{3+} binding<br>2. Transport Fe^{3+} within transmembrane<br>3. Involved in iron ion homeostasis in cells
	LMAN1 protein	6.3	1. Mannose (monosaccharide hexose) binding.<br>2. Endoplasmic reticulum (ER) to golgi body facilitated transport
	Globin C1	9.79	1. Metal binding (binding with Ca^{2+}, Mg^{2+} and Fe^{3+})<br>2. Heme-binding<br>3. Transport oxygen
	Globin B1	6.75	1. Fe^{2+}, Fe^{3+} binding<br>2. Heme binding<br>3. Organic acid binding<br>4. Oxygen binding<br>5. Hemoglobin alpha binding<br>6. Transports oxygen
	Globin A1	7.02	1. Fe^{2+}, Fe^{3+} binding<br>2. Heme binding<br>3. Oxygen binding<br>4. Transports oxygen
	Alpha-1-acid glycoprotein (positive acute phase protein)	4.93	1. Behave as transport protein in blood stream.<br>2. Involved in regulation of immune system.<br>3. Inhibits aggregation of platelets and neutrophils.

	Mutant hemoglobin alpha 1 globin chain	8.08	1. Fe^{2+}, Fe^{3+} binding<br>2. Heme binding<br>3. Oxygen binding<br>4. Transport oxygen<br>5. Transport bicarbonate
	Hemoglobin alpha-1 globin chain	8.08	1. Fe^{2+}, Fe^{3+} binding<br>2. Heme binding<br>3. Oxygen binding<br>4. Transport oxygen<br>5. Transport bicarbonate
	Hemoglobin subunit alpha	8.72	1. Fe^{2+}, Fe^{3+} binding<br>2. Heme binding<br>3. Oxygen binding<br>4. Transport oxygen
	Alpha-2 globin chain	8.72	1. Fe^{2+}, Fe^{3+} binding<br>2. Heme binding<br>3. Oxygen binding<br>4. Transport oxygen<br>5. Transport bicarbonate
	Mutant hemoglobin alpha 2 globin chain	8.72	1. Fe^{2+}, Fe^{3+} binding<br>2. Heme binding<br>3. Oxygen binding<br>4. Transport oxygen
	Lactoferrin	8.70	1. Fe^{2+}, Fe^{3+} binding<br>2. Involved in serine-type endopeptidase activity<br>3. Engender antimicrobial humoral response<br>4. Regulates the production of cytokine
	Beta-globin protein	6.64	1. Heme binding<br>2. Metal ion binding<br>3. Oxygen binding<br>4. Transports oxygen<br>5. Makes haemoglobin (HbA) along with alpha-globin in adults
	Hemoglobin fetal subunit beta	6.75	1. Fe^{2+}, Fe^{3+} binding<br>2. Heme binding<br>3. Organic acid binding<br>4. Oxygen binding<br>5. Hemoglobin alpha binding<br>6. Transports oxygen
	Truncated beta-globin	7.96	1. Heme binding<br>2. Oxygen binding
	Mutant beta-globin	7.92	1. Heme binding<br>2. Oxygen binding<br>3. Transports oxygen
	Beta-globin chain	5.57	1. Fe^{2+}, Fe^{3+} binding<br>2. Heme binding<br>3. Oxygen binding<br>4. Transport oxygen
	Hemoglobin subunit beta	6.25	1. Heme binding<br>2. Oxygen binding<br>3. Transports oxygen
	Hemoglobin beta chain	6.74	1. Fe^{2+}, Fe^{3+} binding<br>2. Heme binding<br>3. Oxygen binding<br>4. Transport oxygen
	Hemoglobin beta chain variant Hb.Sinai-Bel Air	5.41	1. Fe^{2+}, Fe^{3+} binding<br>2. Heme binding<br>3. Oxygen binding

			4. Transport oxygen
	Hemoglobin beta chain variant Hb-I_Toulouse	5.41	1. Fe^{2+}, Fe^{3+} binding<br>2. Heme binding<br>3. Oxygen binding<br>4. Transport oxygen
	Vitamin D-binding protein	5.2	1. Vitamin D binding<br>2. Transport vitamin within transmembrane
**Cell adhesive protein**	cDNA FLJ54111	6.95	1. Ca^{2+} binding<br>2. Collagen binding
**Coagulation protein**	Prothrombin	5.64	1. Non-covalently calcium ions (Ca^{2+}) binding.<br>2. Blood coagulation<br>3. Involved in serine-type endopeptidase activity
**Metabolic Protein**	Adiponectin	5.42	1. Involved in fat metabolism and hormonal activity<br>2. Identical protein binding<br>3. Sialic acid binding<br>4. Signaling receptor binding<br>5. Involved in glucose metabolism<br>6. Involved in the negative regulation of macrophage differentiation and phagocytosis
**Protease inhibitor**	Kininogen-1	6.02	1. Inhibits thiol proteases<br>2. Inhibits the thrombin- and plasmin-induced aggregation of thrombocytes in blood coagulation<br>3. Heparin binding<br>4. Zinc ion binding<br>5. Regulates cell adhesion
	KNG1 protein	6.4	1. Inhibits cysteine-type endopeptidase
	Fetuin-B	8.7	1. Inhibits metalloenzyme, metalloprotease and protease, i.e- inhibits cysteine-type endopeptidase and metallo-endopeptidase.<br>2. Involved in fertilization.
	Alpha-1-antiproteinase	6.05	1. Inhibits protease and serine protease.<br>2. Inhibits trypsin, chymotrypsin and plasminogen activator.<br>3. Identical protein and protease binding.
**Structural Protein**	Keratin, type I cytoskeletal 10	5.13	1. Cross linking of peptides<br>2. Protein heterodimerization and heterotetramerization activity<br>3. Keratinization
	Keratin 1	8.15	1. Involved in structural molecular activity
	KRT6A protein	8.09	1. Involved in the structural molecular activity
	Keratin, type I cytoskeletal 14	5.09	1. Responsing to Zn^{2+}<br>2. Keratin filament binding<br>3. Structural constituent of cytoskeleton<br>4. Keratinization
	Histone H2B	10.32	1. DNA binding<br>2. Involved in protein heterodimerization and nucleosome assembly
**Regulatory Protein**	Junction mediating and regulatory protein p53 cofactor	5.95	1. Actin binding.<br>2. Arp2/3 complex binding<br>3. Involved in transcription coactivator activity.<br>4. Involved in cell cycle arrest.<br>5. Involved in positive regulation of apoptosis.
**Others**	Uncharacterized protein	6.28	1. Fe^{2+}, Fe^{3+} binding<br>2. Heme binding<br>3. Oxygen binding<br>4. Transport oxygen

	Epididymis secretory protein Li 51	5.4	1. Vitamin D binding<br>2. Transports vitamin transmembrane
	Alpha-fetoprotein	4.57	1. Metal (copper, nickel) binding<br>2. Fatty acids binding<br>3. Bilirubin binding<br>4. Cellular protein metabolism<br>5. Progesterone metabolism
	cDNA FLJ95666	5.92	1. Involved in aspirin acetylated lysine<br>2. Bilirubin binding
	cDNA FLJ57154	6.29	1. Helps in heterologous expression
	cDNA FLJ55606	5.84	1. Cysteine-type endopeptidase inhibitor

Table S6. List of identified proteins with low Fe/Mg-CA in 10% FBS along with their isoelectric point (pI) and major biological functions.

Functional classification of protein	Identified Protein	pI	Detailed Function
**Transport protein**	Albumin	5.53	1.Transport<br>2. Systemic calcification inhibitor<br>3. Lipid binding<br>4. Metal binding<br>5. Maintaining intravascular colloid osmotic pressure (COP)<br>6. Dysopsonin
	Mutant hemoglobin alpha 1 globin chain	8.72	1. Fe^{2+}, Fe^{3+} binding<br>2. Heme binding<br>3. Oxygen binding<br>4. Transport oxygen<br>5. Transport bicarbonate
	Hemoglobin alpha-1 globin chain	8.72	1. Fe^{2+}, Fe^{3+} binding<br>2. Heme binding<br>3. Oxygen binding<br>4. Transport oxygen<br>5. Transport bicarbonate
	Alpha-2 globin chain	8.72	1. Fe^{2+}, Fe^{3+} binding<br>2. Heme binding<br>3. Oxygen binding<br>4. Transport oxygen<br>5. Transport bicarbonate
	Hemoglobin subunit alpha piens	8.72	1. Fe^{2+}, Fe^{3+} binding<br>2. Heme binding<br>3. Oxygen binding<br>4. Transport oxygen<br>5. Transport bicarbonate<br>6. Receptor mediated endocytosis<br>7. Regulation of cell death<br>8. Protein heterooligomerization
	Mutant hemoglobin alpha 2 globin chain	8.72	1. Fe^{2+}, Fe^{3+} binding<br>2. Heme binding<br>3. Oxygen binding<br>4. Transport oxygen
	Hemoglobin subunit alpha	8.72	1. Fe^{2+}, Fe^{3+} binding<br>2. Heme binding<br>3. Oxygen binding

			4. Transport oxygen
	Globin C1	9.79	1. Metal binding (binding with Ca^{2+}, Mg^{2+} and Fe^{3+})<br>2. Heme-binding<br>3. Transport oxygen
	Beta-globin protein	6.64	1. Heme binding<br>2. Fe^{2+}, Fe^{3+} binding<br>3. Oxygen binding<br>4. Transports oxygen<br>5. Makes haemoglobin (HbA) along with alpha-globin in adults
	Beta-globin chain	5.57	1. Fe^{2+}, Fe^{3+} binding<br>2. Heme binding<br>3. Oxygen binding<br>4. Transport oxygen
	Truncated beta-globin	7.96	1. Heme binding<br>2. Oxygen binding
	Mutant beta-globin	7.92	1. Heme binding<br>2. Oxygen binding<br>3. Transports oxygen
	Hemoglobin subunit beta	6.25	1. Heme binding<br>2. Oxygen binding<br>3. Transports oxygen
	Hemoglobin beta	5.45	1. Fe^{2+}, Fe^{3+} binding<br>2. Heme binding<br>3. Oxygen binding<br>4. Transport oxygen
	Hemoglobin beta chain variant Hb-I_Toulouse	5.41	1. Fe^{2+}, Fe^{3+} binding<br>2. Heme binding<br>3. Oxygen binding<br>4. Transport oxygen
	Mutant hemoglobin beta chain (Fragment)	6.5	1. Fe^{2+}, Fe^{3+} binding<br>2. Heme binding<br>3. Oxygen binding<br>4. Transport oxygen
	Hemoglobin beta globin chain	6.74	1. Fe^{2+}, Fe^{3+} binding<br>2. Heme binding<br>3. Oxygen binding<br>4. Transport oxygen
	Hemoglobin beta	6.74	1. Fe^{2+}, Fe^{3+} binding<br>2. Heme binding<br>3. Oxygen binding<br>4. Transport oxygen
	Hemoglobin beta chain variant Hb.Sinai-Bel Air	5.41	1. Fe^{2+}, Fe^{3+} binding<br>2. Heme binding<br>3. Oxygen binding<br>4. Transport oxygen
	Alpha-2-HS glycoprotein	5.94	1. Enhance endocytosis<br>2. Ca^{2+} binding<br>3. Post-translational protein modification<br>4. Protein metabolism
	Transthyretin	5.6	1. Thyroid hormone binding<br>2. Identical protein binding<br>3. Transport iron<br>4. Transport thyroxine to the brain<br>5. Cellular protein metabolism<br>6. Neutrophil degranulation<br>7. Retinol metabolism

	Serotransferrin	6.81	1. Fe^{3+} binding<br>2. Bicarbonate binding<br>3. Transport iron
	Transferrin variant	6.68	1. Fe^{3+} binding<br>2. Transport Fe^{3+} within transmembrane<br>3. Involved in iron ion homeostasis in cells
	Transferrin	6.97	1. Fe^{3+} binding<br>2. Transport Fe^{3+} within transmembrane<br>3. Involved in iron ion homeostasis in cells
	Lactotransferrin	8.7	1. Fe^{3+} binding<br>2. Cu^{2+} binding<br>3. Bicarbonate binding<br>4. Involved in serine-type endopeptidase activity<br>5. Engender antimicrobial humoral response<br>6. Regulates the production of cytokine
	Lactoferrin	8.7	1. Fe^{2+}, Fe^{3+} binding<br>2. Involved in serine-type endopeptidase activity<br>3. Engender antimicrobial humoral response<br>4. Regulates the production of cytokine
	Delta globin	7.85	1. Fe^{2+}, Fe^{3+} binding<br>2. Heme binding<br>3. Oxygen binding<br>4. Transport oxygen<br>5. Blood coagulation
	Hbbm fused globin protein	6.17	1. Fe^{2+}, Fe^{3+} binding<br>2. Heme binding<br>3. Oxygen binding<br>4. Transport oxygen
**Coagulation protein**	Prothrombin	5.64	1. Non-covalently calcium ions (Ca^{2+}) binding.<br>2. Blood coagulation<br>3. Involved in serine-type endopeptidase activity
**Cell adhesive protein**	cDNA FLJ54111	6.95	1. Ca^{2+} binding<br>2. Collagen binding
	Proliferation-inducing protein 33	4.71	1. Ca^{2+} binding<br>2. Collagen binding
	C-type lectin domain family 3 member B isoform CRA_a	5.21	1. Carbohydrate binding<br>2. Ossification
**Enzymes**	N-acetyltransferase 5	5.84	1. Involved in transferase activity<br>2. Catalyzes the transfer of acetyl groups from acetyl CoA to arylamines
**Structural protein**	cDNA FLJ56708	8.44	1. Involved in structural molecular activity
	Keratin, type I cytoskeletal 10	5.13	1. Cross linking of peptides<br>2. Protein heterodimerization and heterotetramerization activity<br>3. Keratinization
	Keratin, type I cytoskeletal 28	5.33	1. Involved in structural molecular activity<br>2. Cornification<br>3. keratinization
	Keratin 1	8.15	1. Involved in structural molecular activity
	Keratin, type II cytoskeletal 2	8.07	1.Cytoskeletal protein binding<br>2. Cross linking of peptides<br>3. Structural constituent of cytoskeleton<br>4. Keratinization

**Others**	Protein unc-13 homolog B	5.67	1. Ca^{2+}binding<br>2. Calmodulin binding<br>3. Phospholipid binding<br>4. Diacylglycerol binding<br>5. Intracellular signal transduction<br>6. Presynaptic and synaptic vesicle Exocytosis
	MHC class I antigen	6.64	1. Peptide binding engendered by cytosolic protein degradation via the proteasome<br>2. Exhibit intracellular proteins to cytotoxic T cells<br>3. Function as an inhibitory ligand for natural killer cells
	Alpha-fetoprotein	4.57	1. Metal (copper, nickel) binding<br>2. Fatty acids binding<br>3. Bilirubin binding<br>4. Cellular protein metabolism<br>5. Progesterone metabolism
	cDNA FLJ57154	6.29	1. Helps in heterologous expression
	cDNA FLJ79229	8.10	1. Fe^{2+}, Fe^{3+} binding<br>2. Controls cytokine production<br>3. Involved in antimicrobial humoral response
	Uncharacterized protein	6.28	1. Fe^{2+}, Fe^{3+} binding<br>2. Heme binding<br>3. Oxygen binding<br>4. Transport oxygen
	A-kinase anchor protein	8.84	1. Protein kinase A (PKA) binding<br>2. Specific substrate targeting by means of phosphorylation (through PKA) and dephosphorylation (through phosphatases)
	Zinc finger SWIM domain-containing protein 4	6.47	1. Zn^{2+} binding<br>2. DNA binding
	Capsid scaffolding protein	8.10	1.Identical protein binding<br>2. Involved in nuclear capsid macromolecular assembly<br>3. Involved in serine-type endopeptidase activity
	cDNA FLJ51509	7.42	1.Behaves similar to Alpha-fetoprotein, i.e- takes part in metal, fatty acids and bilirubin binding, involved in cellular protein and progesterone metabolism
	cDNA FLJ55606	5.84	1. Cysteine-type endopeptidase inhibitor

Table S7. List of identified proteins with high Fe/Mg-CA in 10% FBS along with their isoelectric point (pI) and major biological functions.

Functional classification of protein	Identified Protein	pI	Detailed Function
**Transport protein**	Albumin	5.53	1.Transport<br>2. Systemic calcification inhibitor<br>3. Lipid binding<br>4. Metal binding<br>5. Maintaining intravascular colloid osmotic pressure (COP)

	Globin C1	9.79	1. Metal binding (binding with Ca^{2+}, Mg^{2+} and Fe^{3+}).<br>2. Heme-binding<br>3. Transport oxygen.
	Alpha-2 globin chain	8.72	1. Fe^{2+}, Fe^{3+} binding<br>2. Heme binding<br>3. Oxygen binding<br>4. Transport oxygen<br>5. Transport bicarbonate
	Hemoglobin subunit alpha	8.72	1. Fe^{2+}, Fe^{3+} binding<br>2. Heme binding<br>3. Oxygen binding<br>4. Transport oxygen
	Mutant hemoglobin alpha 1 globin chain	8.72	1. Fe^{2+}, Fe^{3+} binding<br>2. Heme binding<br>3. Oxygen binding<br>4. Transport oxygen<br>5. Transport bicarbonate
	Hemoglobin alpha-1 globin chain	8.72	1. Fe^{2+}, Fe^{3+} binding<br>2. Heme binding<br>3. Oxygen binding<br>4. Transport oxygen<br>5. Transport bicarbonate
	Mutant hemoglobin alpha 2 globin chain	8.72	1. Fe^{2+}, Fe^{3+} binding<br>2. Heme binding<br>3. Oxygen binding<br>4. Transport oxygen
	Beta-globin protein	6.64	1. Heme binding<br>2. Makes haemoglobin (HbA) along with alpha-globin in adults
	Alpha-2-HS glycoprotein	5.94	1. Enhance endocytosis<br>2. Ca^{2+} binding<br>3. Post-translational protein modification<br>4. Protein metabolism
	Transthyretin	5.6	1. Thyroid hormone binding<br>2. Identical protein binding<br>3. Transport iron<br>4. Transports thyroxine to the brain<br>5. Cellular protein metabolism<br>6. Neutrophil degranulation<br>7. Retinol metabolism
	Serotransferrin	6.81	1. Fe^{3+} binding<br>2. Bicarbonate binding<br>3. Transport iron
	Transferrin variant	6.68	1. Fe^{3+} binding<br>2. Transport Fe^{3+} within transmembrane<br>3. Involved in iron ion homeostasis in cells
	Transferrin	6.97	1. Fe^{3+} binding<br>2. Transport Fe^{3+} within transmembrane<br>3. Involved in iron ion homeostasis in cells
	Tetranectin	5.8	1. Ca^{2+} binding<br>2. Heparin binding<br>3. Carbohydrate binding<br>*4.* Cellular metabolism<br>5. Bone mineralization<br>6. Ossification<br>7. Platelet degranulation
	cDNA FLJ54839	7.42	1. Behaves similar to Lactotransferrin precursor
	cDNA FLJ53691	6.81	1. Fe^{3+} binding

			2. Transport Fe^{3+} within transmembrane
	Lactotransferrin	8.7	1. Fe^{3+} binding<br>2. Cu^{2+} binding<br>3. Bicarbonate binding<br>4. Involved in serine type endopeptidase activity<br>5. Engender antimicrobial humoral response<br>6. Regulates the production of cytokine
	Lactoferrin	8.7	1. Fe^{2+}, Fe^{3+} binding<br>2. Involved in serine type endopeptidase activity<br>3. Engender antimicrobial humoral response<br>4. Regulates the production of cytokine
	Delta globin	7.85	1. Fe^{2+}, Fe^{3+} binding<br>2. Heme binding<br>3. Oxygen binding<br>4. Transport oxygen<br>5. Blood coagulation
	Hemoglobin subunit gamma-2	6.64	1. Fe^{2+}, Fe^{3+} binding<br>2. Heme binding<br>3. Oxygen binding<br>4. Transport oxygen<br>5. Blood coagulation
	Globin B3	8.67	1. Fe^{2+}, Fe^{3+} binding<br>2. Heme binding<br>3. Hemoglobin alpha binding<br>4. Oxygen binding<br>5. Transport oxygen
	Gamma-G globin	7.2	1. Fe^{2+}, Fe^{3+} binding<br>2. Heme binding<br>3. Oxygen binding<br>4. Transport oxygen
	Hbbm fused globin protein	6.17	1. Fe^{2+}, Fe^{3+} binding<br>2. Heme binding<br>3. Oxygen binding<br>4. Transport oxygen
**Cell adhesive protein**	SPARC-like protein 1	4.73	1. Ca^{2+} binding<br>2.Collagen binding<br>3. Extracellular matrix binding<br>4. Adhession of synaptic membrane<br>5.Cellular protein metabolism
	Proliferation-inducing protein 33	4.71	1. Ca^{2+} binding<br>2. Collagen binding
	C-type lectin domain family 3 member B isoform CRA_a	5.21	1. Carbohydrate binding<br>2. Ossification
	cDNA FLJ54111	6.95	1. Ca^{2+} binding<br>2. Collagen binding
	cDNA FLJ54278	4.83	1. Ca^{2+} binding<br>2. Collagen binding
	cDNA FLJ54387	4.71	1. Ca^{2+} binding<br>2. Collagen binding
	cDNA FLJ55140	4.73	1. Ca^{2+} binding<br>2. Collagen binding

**Coagulation protein**	Prothrombin	5.64	1. Non-covalently calcium ions (Ca^{2+}) binding.<br>2. Blood coagulation<br>3. Involved in serine-type endopeptidase activity
**Structural protein**	Keratin, type II cytoskeletal 2 epidermal	8.07	1.Cytoskeletal protein binding<br>2. Cross linking of peptides<br>3. Structural constituent of cytoskeleton<br>4. Keratinization
	Keratin, type II cytoskeletal 5	7.58	1. Scaffold protein binding<br>2. Structural constituent of cytoskeleton<br>3. Keratinization
	Keratin, type I cytoskeletal 14	5.09	1. Responsing to Zn^{2+}<br>2. Keratin filament binding<br>3. Structural constituent of cytoskeleton<br>4. Keratinization
	Keratin 77	5.8	1. Involved in structural integrity of epithelial cells<br>2. Involved in structural molecular activity
	Keratin 1	8.15	1. Significant for structural integrity of a complex or its assembly within or outside a cell.
	Keratin, type I cytoskeletal 9	5.14	1. Keratin filament binding<br>2. Structural constituent of cytoskeleton<br>3. Keratinization<br>4. Epidermis development<br>5. Intermediate filament organization
	Keratin, type I cytoskeletal 10	5.13	1. Cross linking of peptides<br>2. Protein heterodimerization and heterotetramerization activity<br>3. Structural constituent of epidermis<br>4. Keratinization
**Others**	cDNA FLJ57154	6.29	1. Helps in heterologous expression
	cDNA FLJ58606	7.82	1. Helps in heterologous expression
	Protein S100-A7A	6.89	1. Ca^{2+} binding<br>2. Transition metal binding<br>3. Involved in protein self-association
	A-kinase anchor protein	8.84	1. Protein kinase A (PKA) binding<br>2. Specific substrate targeting by means of phosphorylation (through PKA) and dephosphorylation (through phosphatases)
	Epididymis luminal protein 110	7.8	1. Fe^{2+}, Fe^{3+} binding<br>2. Involved in serine type endopeptidase action<br>3. Engender antimicrobial humoral response<br>4. Regulates the production of cytokine
	Zinc finger SWIM domain-containing protein 4	6.47	1. Zn^{2+} binding<br>2. DNA binding
	Alpha-fetoprotein	4.57	1. Metal (copper, nickel) binding<br>2. Fatty acids binding<br>3. Bilirubin binding<br>4. Cellular protein metabolism<br>5. Progesterone metabolism
	Protein unc-13 homolog B	5.67	1. Ca^{2+} binding<br>2. Calmodulin binding<br>3. Phospholipid binding<br>4. Diacylglycerol binding<br>5. Intracellular signal transduction<br>6. Presynaptic and synaptic vesicle exocytosis

Table S8: Protein corona profile of CA in 10% FBS in terms of their molecular weight (Da), -10lgP and coverage (%).

Functional classification of protein	Identified Protein	Molecular Weight (Da)	-10lgP	Coverage (%)
**Transport Protein**	ALB protein	69294	260.46	80
	Serum Albumin	69227	207.46	40
	Alpha-2-HS glycoprotein	39325	121.73	11
	cDNA FLJ51509	54207	107.1	15
	Transthyretin	15872	80.92	28
	Serotransferrin	77064	92.84	7
	Transferrin variant	77080	92.84	7
	Transferrin	76960	92.84	7
	LMAN1 protein	57549	21.54	1
	Globin C1	19232	78.40	23
	Globin B1	16071	33.31	7
	Globin A1	15954	33.31	7
	Alpha-1-acid glycoprotein (positive acute phase protein)	23512	37.40	6
	Mutant hemoglobin alpha 1 globin chain	10753	78.40	40
	Hemoglobin alpha-1 globin chain	10783	78.40	40
	Hemoglobin subunit alpha	20146	78.40	28
	Alpha-2 globin chain	15258	78.40	28
	Mutant hemoglobin alpha 2 globin chain	15258	78.40	28
	Lactoferrin	78357	69.91	4
	Beta-globin protein	4550	59.21	56
	Hemoglobin fetal subunit beta	15859	33.31	7
	Truncated beta-globin	6998	59.21	37
	Mutant beta-globin	9689	59.21	26
	Beta-globin chain	11476	59.21	26
	Hemoglobin subunit beta	9670	59.21	26
	Hemoglobin beta chain	11023	59.21	23

	Hemoglobin beta chain variant Hb.Sinai-Bel Air	11548	59.21	22
	Hemoglobin beta chain variant Hb-I_Toulouse	11505	59.21	22
	Vitamin D-binding protein	53021	47.50	1
**Cell adhesive protein**	cDNA FLJ54111	63437	92.84	8
**Coagulation protein**	Prothrombin	35932	39.89	3
**Metabolic Protein**	Adiponectin	26133	39.64	4
**Protease inhibitor**	Kininogen-1	71957	43.16	2
	KNG1 protein	33055	43.16	3
	Fetuin-B	42663	49.43	11
	Alpha-1-antiproteinase	46104	118.31	18
**Structural Protein**	Keratin, type I cytoskeletal 10	54848	86.65	14
	Keratin 1	66126	101.91	11
	KRT6A protein	60045	62.63	4
	Keratin, type I cytoskeletal 14	51561	72.04	8
	Histone H2B	13926	20.26	7
**Regulatory Protein**	Junction mediating and regulatory protein p53 cofactor	109383	21.54	1
**Others**	Uncharacterized protein	18931	59.21	13
	Epididymis secretory protein Li 51	52964	47.50	1
	Alpha-fetoprotein	68678	107.1	12
	cDNA FLJ95666	69393	206.8	39
	cDNA FLJ57154	50756	107.10	16
	cDNA FLJ55606	46627	121.73	9

Table S9: Protein corona profile of low Fe/Mg-CA in 10% FBS in terms of their molecular weight (Da), -10lgP and coverage (%).

Functional classification of protein	Identified Protein	Molecular Weight (Da)	-10lgP	Coverage (%)
**Transport protein**	Albumin	66531	180.69	36
	Mutant hemoglobin alpha 1 globin chain	10753	95.34	47
	Hemoglobin alpha-1 globin chain	10783	95.34	47
	Alpha-2 globin chain	15258	95.34	33
	Hemoglobin subunit alpha piens	15258	95.34	33
	Mutant hemoglobin alpha 2 globin chain	15258	95.34	33
	Hemoglobin subunit alpha	20146	95.34	33
	Globin C1	19232	95.34	27
	Beta-globin protein	4550	57.88	56
	Beta-globin chain	11476	62.94	22
	Truncated beta-globin	6998	57.88	37
	Mutant beta-globin	9689	62.94	26
	Hemoglobin subunit beta	9670	57.88	26
	Hemoglobin beta chain	11023	57.88	23
	Hemoglobin beta chain variant Hb-I_Toulouse	11505	62.94	22
	Mutant hemoglobin beta chain (Fragment)	11501	57.88	22
	Hemoglobin beta globin chain	11494	57.88	22
	Hemoglobin beta	11482	62.94	22
	Hemoglobin beta chain variant Hb.Sinai-Bel Air	11548	57.88	22
	Alpha-2-HS glycoprotein	39325	62.15	13
	Transthyretin	15872	88.27	44
	Serotransferrin	77064	77.82	5
	Transferrin variant	77080	77.82	5
	Transferrin	76960	77.82	5
	Lactotransferrin	76626	55.45	3
	Lactoferrin	77981	55.45	3
	Delta globin	16055	48.92	7
	Hbbm fused globin protein	10928	40.72	10
**Coagulation protein**	Prothrombin	35932	37.17	3
**Cell adhesive protein**	cDNA FLJ54111	63437	71.87	6
	Proliferation-inducing protein 33	75230	72.24	5
	C-type lectin domain family 3 member B isoform CRA_a	22537	48.09	10
**Enzymes**	N-acetyltransferase 5	18857	24.33	4
**Structural protein**	cDNA FLJ56708	18049	62.03	15
	Keratin, type I cytoskeletal 10	58827	154.11	34
	Keratin, type I cytoskeletal 28	50567	59.40	5

	Keratin 1	66126	145.46	22
	Keratin, type II cytoskeletal 2	65433	103.32	13
**Others**	Protein unc-13 homolog B	180679	21.64	1
	MHC class I antigen	12392	23.42	4
	Alpha-fetoprotein	68678	70.56	6
	cDNA FLJ57154	50756	54.72	7
	cDNA FLJ79229	73170	68.49	3
	Uncharacterized protein	18931	57.88	13
	A-kinase anchor protein	161184	21.18	1
	Zinc finger SWIM domain-containing protein 4	99649	29.84	1
	Capsid scaffolding protein	66941	21.94	1
	cDNA FLJ51509	54207	70.56	8
	cDNA FLJ55606	46627	62.15	11

Table S10: Protein corona profile of high Fe/Mg-CA in 10% FBS in terms of their molecular weight (Da), -10lgP and coverage (%).

Functional classification of protein	Identified Protein	Molecular Weight (Da)	-10lgP	Coverage (%)
**Transport protein**	Albumin	66531	158.89	27
	Globin C1	19232	90.79	23
	Alpha-2 globin chain	15258	86.99	28
	Hemoglobin subunit alpha	20146	86.99	28
	Mutant hemoglobin alpha 1 globin chain	10753	86.99	40
	Hemoglobin alpha-1 globin chain	10783	86.99	40
	Mutant hemoglobin alpha 2 globin chain	15258	86.99	28
	Beta-globin protein	2104	40.72	59
	Alpha-2-HS glycoprotein	39325	55.71	9
	Transthyretin	15872	47.21	24
	Serotransferrin	77064	71.87	5
	Transferrin variant	77080	71.87	5
	Transferrin	76960	71.87	5
	Tetranectin	17794	48.09	13
	cDNA FLJ54839	54207	55.45	7
	cDNA FLJ53691	74832	71.87	5
	Lactotransferrin	76626	55.45	3
	Lactoferrin	77981	55.45	3
	Delta globin	16055	48.92	7
	Hemoglobin subunit gamma-2	15319	40.72	7
	Globin B3	16203	40.72	7

	Gamma-G globin	16969	40.72	6
	Hbbm fused globin protein	10928	40.72	10
**Cell adhesive protein**	SPARC-like protein 1	75208	72.24	5
	Proliferation-inducing protein 33	75230	72.24	5
	C-type lectin domain family 3 member B isoform CRA_a	22537	48.09	10
	cDNA FLJ54111	63437	71.87	6
	cDNA FLJ54278	58507	72.24	7
	cDNA FLJ54387	64141	72.24	6
	cDNA FLJ55140	55519	72.24	7
**Coagulation protein**	Prothrombin	35932	35.09	3
**Structural protein**	Keratin, type II cytoskeletal 2 epidermal	65433	131.06	13
	Keratin, type II cytoskeletal 5	62378	97.24	11
	Keratin, type I cytoskeletal 14	51562	95.71	13
	Keratin 77	61802	81.98	5
	Keratin 1	66126	232.15	40
	Keratin, type I cytoskeletal 9	62064	183.04	33
	Keratin, type I cytoskeletal 10	58827	172.78	37
**Others**	cDNA FLJ57154	50756	54.72	7
	cDNA FLJ58606	91670	29.84	1
	Protein S100-A7A	11305	36.53	11
	A-kinase anchor protein	161184	21.18	1
	Epididymis luminal protein 110	78182	55.45	3
	Zinc finger SWIM domain-containing protein 4	99649	29.84	1
	Alpha-fetoprotein	68678	54.72	5
	Protein unc-13 homolog B	180679	21.64	1
